# Genomic classification and antimicrobial resistance profiling of *Streptococcus pneumoniae* and *Haemophilus influenzae* isolates associated with paediatric otitis media and upper respiratory infection

**DOI:** 10.1186/s12879-023-08560-x

**Published:** 2023-09-13

**Authors:** Briallen Lobb, Matthew C. Lee, Christi L. McElheny, Yohei Doi, Kristin Yahner, Alejandro Hoberman, Judith M. Martin, Jeremy A. Hirota, Andrew C. Doxey, Nader Shaikh

**Affiliations:** 1 Department of Biology and Waterloo Centre for Microbial Research, University of Waterloo, Waterloo, ON Canada; 2 University of Pittsburgh School of Medicine, Children’s Hospital of Pittsburgh of UPMC, Division of General Academic Pediatrics, Pittsburgh, USA; 3 Division of Infectious Diseases, University of Pittsburgh School of Medicine, Pittsburgh, PA USA; 4 Department of Medicine, McMaster University, Hamilton, ON Canada; 5 Firestone Institute for Respiratory Health, St. Joseph’s Hospital, Hamilton, ON Canada; 6 Department of Medicine, University of British Columbia, Vancouver, BC Canada

**Keywords:** Acute otitis media, Upper respiratory infection, *Streptococcus pneumoniae*, *Haemophilus influenzae*, Antibiotics, Antimicrobial resistance, Pathogens, Genomic surveillance

## Abstract

Acute otitis media (AOM) is the most common childhood bacterial infectious disease requiring antimicrobial therapy. Most cases of AOM are caused by translocation of *Streptococcus pneumoniae* or *Haemophilus influenzae* from the nasopharynx to the middle ear during an upper respiratory tract infection (URI). Ongoing genomic surveillance of these pathogens is important for vaccine design and tracking of emerging variants, as well as for monitoring patterns of antibiotic resistance to inform treatment strategies and stewardship.

In this work, we examined the ability of a genomics-based workflow to determine microbiological and clinically relevant information from cultured bacterial isolates obtained from patients with AOM or an URI. We performed whole genome sequencing (WGS) and analysis of 148 bacterial isolates cultured from the nasopharynx (*N* = 124, 94 AOM and 30 URI) and ear (*N* = 24, all AOM) of 101 children aged 6–35 months presenting with AOM or an URI. We then performed WGS-based sequence typing and antimicrobial resistance profiling of each strain and compared results to those obtained from traditional microbiological phenotyping.

WGS of clinical isolates resulted in 71 *S. pneumoniae* genomes and 76 *H. influenzae* genomes. Multilocus sequencing typing (MSLT) identified 33 sequence types for *S. pneumoniae* and 19 predicted serotypes including the most frequent serotypes 35B and 3. Genome analysis predicted 30% of *S. pneumoniae* isolates to have complete or intermediate penicillin resistance. AMR predictions for *S. pneumoniae* isolates had strong agreement with clinical susceptibility testing results for beta-lactam and non beta-lactam antibiotics, with a mean sensitivity of 93% (86–100%) and a mean specificity of 98% (94–100%). MLST identified 29 *H. influenzae* sequence types. Genome analysis identified beta-lactamase genes in 30% of *H. influenzae* strains, which was 100% in agreement with clinical beta-lactamase testing. We also identified a divergent highly antibiotic-resistant strain of *S. pneumoniae*, and found its closest sequenced strains, also isolated from nasopharyngeal samples from over 15 years ago.

Ultimately, our work provides the groundwork for clinical WGS-based workflows to aid in detection and analysis of *H. influenzae* and *S. pneumoniae* isolates.

**Supplementary Information:**

The online version contains supplementary material available at 10.1186/s12879-023-08560-x.

## Background

During viral upper respiratory infections (URI) [1, 2] bacteria present in the nasopharynx can seed the sinuses or middle ear, causing sinusitis [3, 4] or acute otitis media (AOM) [5, 6], respectively. *Streptococcus pneumoniae* and *Haemophilus infuenzae* are the two most frequently observed pathogens in children with AOM [7, 8]. AOM is commonly observed in children aged 6–24 months, affecting approximately 60% of all children at some point in their lifetime [8]. AOM is diagnosed on the basis of an abnormal otoscopic exam in a symptomatic child. Symptoms may include otalgia, fever, and fussiness. Coinfections involving multiple pathogens (bacterial or viral) are not unusual [9]. AOM is the number one indication for antibiotic use in children. Antibiotic resistance among pathogens causing AOM is an increasing concern [8, 10–13]. Proper microbiological identification of AOM pathogens and detection of their antimicrobial susceptibility profile is important for ongoing surveillance, diagnosis, and antimicrobial stewardship [14, 15].

*H. influenzae* is a small gram-negative bacterium in the class Gammaproteobacteria which is often found as a commensal in the nasopharynx. In susceptible populations such as young children, the elderly, and immunocompromised, it can cause numerous opportunistic infections including pneumonia, sinusitis, bronchitis, and otitis media [14]. While *H. influenzae* type B (Hib) was once a major cause of invasive disease worldwide, this has been decreased in regions where Hib vaccination programs have been widely administered [15]. Today, in these regions, non-typeable *H. influenzae* (NTHi) strains have become the common cause of invasive disease [16, 17]. In addition, the global increase of antibiotic-resistant *H. influenzae* strains represent an ongoing threat [18]. These strains of *H. influenzae*, which often carry *bla* (beta-lactamase) genes, possess varying degrees of resistance to common beta-lactam antibiotics including ampicillin [19].

*S. pneumoniae* is a gram-positive organism in the class Bacilli [20]. Similar to *H. influenzae*, it is also commonly found in the respiratory tract of healthy individuals as a commensal organism but is an opportunistic pathogen that can cause a wide range of infections including pneumonia, bronchitis, sinusitis, meningitis, and otitis media. Infections due to *S. pneumoniae* are thought to result in over one million deaths of children annually [21]. Today, over 100 serotypes of *S. pneumoniae* have been identified based largely on structural variation in the capsule [22]. Some strains are more likely to be associated with invasive disease, and these have been preferentially selected historically for inclusion in pneumococcal vaccines [23]. Current pneumococcal conjugate vaccines (e.g., PCV13, PCV15, and PCV20) target up to 20 serotypes, but do not have complete coverage of circulating serotypes associated with infection of young children in western countries [21, 23, 24]. Due to vaccine suppression of certain serotypes, non-vaccine strains can become more common in a population, such as the emergence of serotype 35B among children in the United States [25–30]. The emergence of serotype 35B is also an example that highlights the capability of ongoing genetic adaptation in *S. pneumoniae*, and its ability to acquire patterns of unique multidrug resistance that may be exacerbated by sub-optimal antimicrobial stewardship [25].

While traditional phenotyping and PCR / multilocus sequence typing (MLST)-based methods are often used for clinical pathogen diagnostics and molecular epidemiology, whole genome sequencing (WGS) -based workflows can provide a comprehensive pathogen phylogenetic affiliation, gene content, and predict resistance genes or mutations. WGS-based workflows offer high-resolution pathogenomic profiling and have been critical for genomic epidemiological studies of *H. influenzae* [31, 32] and *S. pneumoniae* [33–35]. WGS also have several advantages over traditional approaches including the ability to determine the taxonomic identity of a pathogen more accurately or identify novel genes and genomic features that are not tested for using traditional diagnostic approaches. The community platform, Pathogenwatch is one such tool that enables automated MLST and prediction of antimicrobial resistance (AMR) profiles from uploaded genomes [36]. Application of WGS technology with AMR detection approaches is very likely to become an essential tool for future antimicrobial stewardship programs.

In the present study, we aimed to assess the effectiveness of a genomics-based workflow for detailed identification of bacterial pathogens associated with AOM and identify AMR genes. We obtained 148 total isolates from the nasopharynx (*N* = 124) and middle ear (*N* = 24) of children aged 6–35 months presenting with AOM (*N* = 93 nasopharynx and *N* = 24 middle ear) or URI (*N* = 31 nasopharynx). Clinical isolates of *S. pneumoniae* (from this point forward referred to as “SPN”) and *H. influenzae* (from this point forward referred to as “HFLU”) were cultured, sequenced, and analyzed using a bioinformatic pipeline powered by Pathogenwatch, CARD, and several other common tools. We then evaluated the phylogenetic diversity of the strains identified, and the extent that WGS-based predictions matched clinically determined AMR phenotypes. Our work on URI and AOM outlines a WGS-based workflow that can be used in future studies of clinical HFLU and SPN isolates to support genomic surveillance efforts.

## Methods

### Description of the cohort

Between October 2019 and June 2021, we prospectively enrolled symptomatic children aged 6–35 months diagnosed with AOM, as well as children who had no AOM but presented with an upper respiratory tract infection. This totaled 150 children, however, three children were enrolled twice (1020, 1027, and 1034) due to repeat infections, making the total number of enrollments 153. Children were recruited from two primary-care offices, one express care center affiliated with the Children’s Hospital of Pittsburgh, and the Otolaryngology Department of the Children’s Hospital of Pittsburgh. AOM was defined by the presence of (1) acute symptoms accompanied by middle-ear effusion and moderate/marked tympanic membrane (TM) bulging, or slight bulging accompanied by either otalgia or marked TM erythema, or (2) acute symptoms accompanied by rupture of a previously intact TM and purulent otorrhea for < 48 h. All children with AOM were treated with guideline concordant antibiotics, while children without AOM were not. Clinician decision and parental consent based on recurrent AOM and/or severe symptoms led to 24 children receiving tympanocentesis. We excluded children from the study with underlying conditions that could affect the course of AOM or upper respiratory infection (e.g., immunodeficiency, chronic perforation of the TM, craniofacial abnormalities). Except for children undergoing tympanocentesis, we excluded children who had received antibiotics within 96 h of enrollment. We collected nasopharyngeal (NP) swabs from all children at the time of diagnosis. For children undergoing tympanocentesis (*N* = 24) and from children with ruptured ear drums (*N* = 13), we also collected middle ear fluid (MEF). From the 13 children with ruptured ear drums, MEF was extracted with suction. NP and MEF swabs were sent to the Clinical Microbiological Laboratory at the Children’s Hospital of Pittsburgh in liquid Amies transport media on the same day as collection.

### Sample processing and bacterial culturing

We used the NP swab or the MEF to inoculate 5% sheep blood agar and chocolate agar plates. We incubated plates for 48 h at 37 °C with 5% CO_2_. SPN and HFLU were identified using standard microbiological techniques. 100 NP samples and 22 MEF samples contained SPN or HFLU, from a total of 101 children (one MEF sample did not have any SPN/HFLU isolated from its paired NP sample). Antibiotic susceptibility testing was performed for SPN isolates on a Vitek 2 (bioMérieux, Inc., Durham, NC) system and interpreted according to current CLSI guidelines [37] (all test results available in Additional File 1: Table S1). HFLU isolates were tested for beta-lactamase production using a Cefinase disc test (Additional File 1: Table S1). If culture positive for HFLU or SPN, colonies were stored in TSB with glycerol. The organism stored in glycerol was regrown and genomic DNA was extracted using the DNeasy Blood and Tissue Kit (Qiagen, Hilden, Germany). The extracted DNA was sent for WGS. Culturing of isolates, beta-lactamase and clinical susceptibility testing was done by the Clinical Microbiology Laboratory.

### WGS, pre-processing and genome assembly

Sample libraries were prepared using the Illumina DNA Prep kit and IDT 10 bp UDI indices, and sequenced on an Illumina NextSeq 2000, producing 2 × 151 bp reads. Demultiplexing, quality control and adapter trimming was performed with bcl-convert (v3.9.3). Raw reads from 148 sequenced clinical isolates were pre-processed using fastp (https://github.com/OpenGene/fastp), which performs adaptor removal, quality filtering and trimming of sequences. Following pre-processing, genomes were assembled using the SPAdes algorithm [38] with the –isolate option. Kraken 2 v2.1.2 [39] was used to taxonomically profile the assembled contigs against the PlusPF database from May 17, 2021 which includes archaea, bacteria, viral, plasmid, human, protozoa, and fungi. Contigs that did not match the expected organism (SPN or HFLU) were removed. The filtered contigs were then analyzed using Pathogenwatch v12.5.3. MLST was performed using PubMLST (https://pubmlst.org/) on all SPN and HFLU clinical isolates, and serotyping was performed on SPN clinical isolates using SeroBA v1.01 [40]. Typing based on the capsule biosynthesis locus for HFLU isolates was done with hicap v1.0.3 [41].

### Subtyping and AMR prediction

For verified SPN clinical isolates, AMR profiles were predicted using Pathogenwatch’s (v12.3.0) AMR and SPN-PBP-AMR prediction modules. The AMR prediction module uses BLASTN to scan genomes for matches to pathogen-specific AMR gene libraries based on CARD (McMaster University—https://card.mcmaster.ca/) [36], ResFinder (https://cge.cbs.dtu.dk/services/ResFinder/), and the NCBI, which can be found here: https://gitlab.com/cgps/pathogenwatch/amr-libraries. An SPN specific library was used for our analysis. The SPN-PBP-AMR prediction module uses the pbp1A, pbp2B and pbp2X genes to predict the minimum inhibitory concentration of beta-lactams [42]. Non-meningitis minimum inhibitory concentration interpretations were used for comparison. For verified HFLU clinical isolates, beta-lactamase gene presence was determined with the Resistance Gene Identifier v5.2.1 against the CARD v3.1.4 at the “Perfect” and “Strict” detection paradigms. CARD contains 220 beta-lactamase gene families. To determine sensitivity and specificity between predicted and clinical AMR predictions, “resistant” and “intermediate” classifications were considered AMR “positive” and “susceptible” was considered AMR “negative”.

### Phylogenomic analysis

Core genome alignments of 71 verified SPN isolates and 76 verified HFLU isolates and their single nucleotide polymorphisms (SNP) profiles were constructed using Snippy v4.6.0 (https://github.com/tseemann/snippy). Reads were aligned to the genome of SPN strain D39V (NCBI accession # NZ_CP027540.1) and HFLU strain 65290_NP_Hi3 (NCBI accession # NZ_QWLX01000001), respectively, as references. SPN strain D39V has been used as a reference genome in previous analyses [43], as has a previous pediatric clinical isolate HFLU strain 65290_NP_Hi3 [44, 45]. A phylogenetic tree was then constructed using RAxML-pthreads v8.2.12 [46] using the autoMRE and GTRGAMMA settings. For visualization, we used the ggTree package v3.2.1 [47] and R version 4.1.1 to map subtypes, serotypes, AMR profiles, beta-lactamase profiles, and clinical metadata onto the phylogenetic trees.

### Analysis of SPN isolate 1001 and S. mitis isolate 1015

All genomes available for these species were downloaded from NCBI-Genbank on Feb. 10, 2022. Average nucleotide identities were obtained between the respective isolate, and the other strains for their identified species using FastANI v1.3 [48]. Strains with top average nucleotide identities to the isolate were chosen to create an alignment using Snippy v4.6.0. For the SPN isolate 1001*,* SPN strain D39V was once again chosen as a reference. For the *S. mitis* isolate 1015, the *S. mitis* strain NCTC 12261 (NCBI accession # NZ_CP028414.1) was chosen as a closely related fully sequenced reference. The trees were made with RAxML-pthreads v8.2.12 [46] using the autoMRE and GTRGAMMA settings. As before, the trees were visualized with the ggtree package v3.2.1 and R version 4.1.1. Pathogenwatch v16.0.0 was used to get serotyping, sequence typing, and AMR prediction for the SPN isolates. VFanalyzer against the Virulence Factor Database [49] was used to identity virulence factors in the isolates and closely related strains on Mar. 24 – Apr. 2, 2022.

## Results and discussion

### Clinical cohort and sampling

**Fig. 1:**
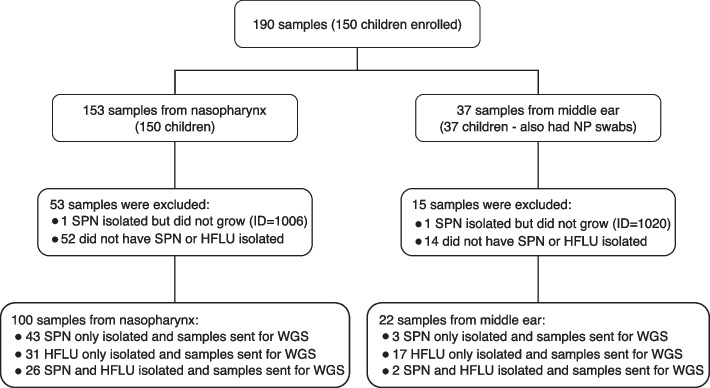
Flow chart of clinical cohort. 100 NP + 22 MEF = 122 samples (101 children). Of the 37 middle ear samples, 35.1% were obtained from perforated TM/suction and 64.9% from tympanocentesis

We enrolled 150 children into the study with AOM or without AOM but presenting with an URI (see Fig. 1 for a flow chart), obtaining nasopharyngeal (NP) swabs from all enrolled, and 37 additional middle ear fluid (MEF) samples from some of the children with AOM. A total of 122 samples from 101 children were positive for HFLU and/or SPN based on culture-based testing (95 samples from children with AOM and 27 samples from children with an URI). Nasal colonization rates for SPN (54%) and HFLU (48%) were higher but comparable to those reported in a previous study on children with AOM from this region (50% for SPN and 29% for HFLU) [50]. The demographic and clinical characteristics of these 101 children are shown in Table 1, and additional metadata is included in Additional File 1: Table S1. In the clinical laboratory, 30% of the SPN isolates were non-susceptible to penicillin, and 30% of the HFLU isolates were beta-lactamase producers. WGS of all isolates resulted in 72 genomes suspected to be SPN and 76 genomes suspected to be HFLU.

**Table 1:** Demographic and clinical characteristics of the cohort (*N* = 101 children)

Age, months		
Mean (SD)	17.0 (8.9)	
Range	[6.0, 35.0]	
Sex		
Female	39	39%
Male	62	61%
Hispanic		
Not Hispanic	96	95%
Hispanic	5	5%
Race		
White	41	41%
Black	49	49%
Multiracial	8	8%
Other	3	3%
Health insurance		
Private	39	39%
Public	61	61%
Child attends school/daycare		
No	37	37%
Yes	63	63%
Diagnosis at time of sample collection (cohort)		
AOM with ruptured TM or requiring tympanocentesis	34	34%
AOM	40	40%
URI	27	27%

### WGS analysis of SPN isolates

The 72 suspected SPN isolates correspond to NP swab and MEF samples from 66 children aged 6–35 months (Additional File 1: Table S1). Following WGS sequencing, we assembled 72 genomes (see Methods). We then performed further quality filtering of the assembled genomes to remove potential contaminant contigs (Additional File 1: Table S2). A total of 71/72 genomes (98.6%) were confirmed as SPN based on taxonomic profiling and MLST analysis. However, one genome (ID 1015) was identified as the related species, *S. mitis*. This organism is also a human pathogen and is occasionally misidentified as SPN based on traditional microbiological methods [51]. BLASTN analysis of contigs against the NCBI nr database confirmed *S. mitis* strains as the closest matches with average nucleotide identities around 95%, suggesting that clinical isolate 1015 is a potentially novel strain of *S. mitis* (Additional File 2: Figure S1).

**Fig. 2:**
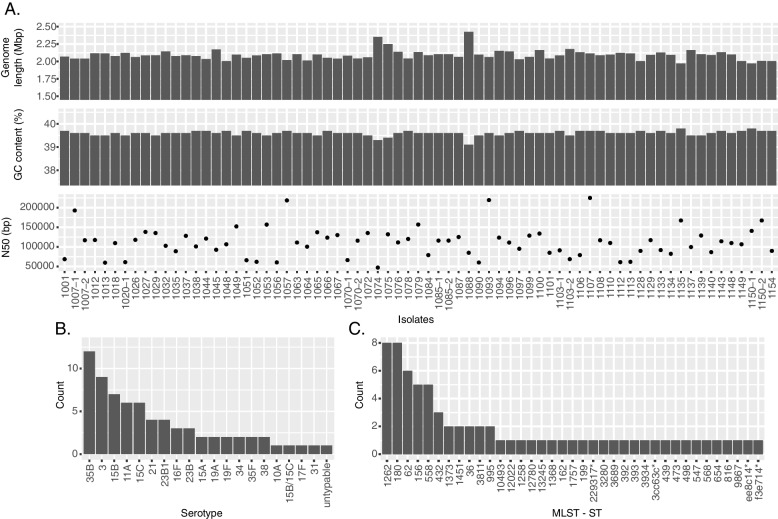
WGS assembly statistics and MLST for 71 clinical SPN isolates. **A** Assembly statistics come from Pathogenwatch (Additional File 1: Table S3). Isolates with “-1” or no “-” came from NP swabs while isolates with “-2” came from MEF samples. **B** Frequency of predicted serotypes for the SPN isolates. **C** Frequency of the MLST sequence types for the SPN isolates. MLST classifications considered “untypable” by MLST are indicated by the first six characters of their MLST classification followed by an asterisk. See Additional File 1: Table S4 for full classification strings

The final assembled SPN genomes had an average total size of 2.09 Mbp, a N50 of 111,808.5 bp, an average contig number of 149.6, and an average GC content of 39.6% (Fig. 2A, Additional File 1: Table S3). The genome size and GC content are consistent with that expected for SPN strains (NCBI SPN complete genome averages are 2.11 +—0.06 Mbp and 39.69 +—0.11%).

#### Phylogenetics and strain typing of SPN isolates

Next, we performed strain typing using the genomic information of each clinical isolate. This was performed in two ways: we used MLST to assign SPN sequence types, and we also used the SeroBA tool [40] to predict the serotype (Additional File 1: Table S4). A breakdown of the typing results is shown in Fig. 2B and C. Among the 71 isolates, we detected 33 different MLST sequence types, revealing a considerable diversity of SPN strains. Four were untypable by MLST. Nineteen different serotypes were predicted for the isolates and only one was untypable. The most common serotypes were 35B (12 isolates) and 3 (9 isolates).

**Fig. 3:**
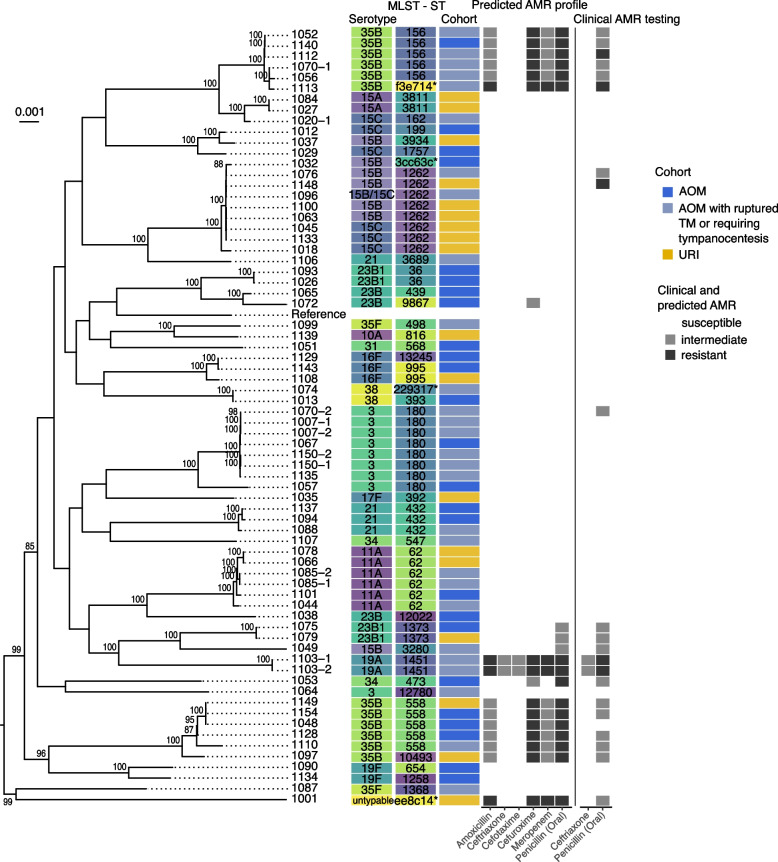
Genome-based phylogeny of SPN strains. Created with 71 SPN strains including strain D39V (NCBI accession # NZ_CP027540.1) as a reference. The mid-point rooted tree is visualized with the ggtree() package in R, and bootstrap support values greater than 80 are indicated above each applicable node in the tree. On the right of the tree, the MLST sequence type, the predicted serotype, sample cohort, Pathogenwatch SPN-PBP-AMR predicted AMR resistance profile, and the clinically tested penicillin susceptibility corresponding to each isolate is shown (see Additional File 1: Table S4 for data including the minimum inhibitory concentration values upon which both the predicted and tested susceptibility is based). Isolates with “-1” or no “-” came from NP while isolates with “-2” came from MEF samples. Colours for the serotype and MLST sequence type columns are just to help differentiate the types. Note: the sequence typing, resistance profile, and clinical metadata for the reference strain was left blank

We then constructed a genome-based phylogenomic tree of the 71 SPN strains using SPN D39V as a reference genome. The genome-based phylogeny reveals a considerable diversification of lineages, consistent with the typing results but with no discernable clustering of sample cohort (Fig. 3).

MLST sequence types and predicted serotypes were mapped onto the phylogenetic tree (Fig. 3). Both typing schemes were highly congruent with the structure of the tree, as specific MLST types and serotypes showed clade-specific patterns. For example, SPN strains from sequence types 1262, 1373, 1451, 36, 62, 432, 180, 156, 3811, and 558, all clustered distinctly into their own groups. In general, predicted serotypes were also congruent with the phylogeny, but some serotypes were distributed across more than one clade (e.g., ST 23B, 35B).

We then compared the genomes of four sets of paired samples (patient IDs 1007, 1103, 1085, 1150) collected from NP swab and MEF from the same individual. For three of these individuals, the strains collected from the NP swab and MEF were virtually identical based on genomic comparison, and clustered as neighbors in the genome-based phylogenetic tree as has been reported in previous studies [52] (Fig. 3). In one individual, however, the NP swab SPN isolate (1070–1) was phylogenetically distinct from the MEF sample isolate (1070–2).

#### AMR profiling of SPN isolates and comparison with clinical results

Next, we bioinformatically predicted AMR profiles of all 71 isolates, initially focusing on a subset of beta-lactam antibiotics including penicillin and ceftriaxone for which clinical testing results were available. To predict susceptibility against these antibiotics, we used the SPN-PBP-AMR method, which assigns minimum inhibitory (MIC) concentrations to antimicrobials and infers resistance phenotypes based on analysis of the *pbp1A*, *pbp2B* and *pbp2X* genes [42]. We then mapped predicted beta-lactam susceptibility profiles onto the SPN phylogenetic tree alongside their clinically measured penicillin susceptibility profiles using oral MIC breakpoints (Fig. 3). In addition to these beta-lactam antibiotics, we predicted susceptibility profiles for several non-beta-lactam antibiotics, including three (tetracycline, erythromycin, and clindamycin) for which clinical testing data was available. Raw data for susceptibility predictions are included in Additional File 1: Table S5.

We then compared the WGS-based predicted resistance profiles with clinically measured susceptibility results for the five antibiotics (penicillin, ceftriaxone, tetracycline, erythromycin, and clindamycin) (Table 2). The WGS-predicted resistance profiles show excellent agreement with clinical testing results. For example, based on the penicillin oral breakpoints for SPN, we achieved a sensitivity and specificity of 86% and 98%, respectively. Eighteen isolates had both a predicted penicillin resistance and a positive clinical test for penicillin resistance. Three isolates, 1070–2, 1076, and 1148, had no predicted resistance to penicillin but demonstrated clinical resistance to it and one isolate, 1048, had predicted resistance without any tested resistance. For the remaining four antibiotics, we also obtained excellent agreement between WGS-based susceptibility predictions and results from traditional testing with a mean sensitivity of 95% and a mean specificity of 98% (Table 2).

**Table 2:** Sensitivity and specificity of predicted versus clinically-tested AMR

	**Penicillin (Oral)**	**Ceftriaxone**	**Tetracycline**	**Erythromycin**	**Clindamycin**
True positives	18	2	8	22	7
Positives	21	2	8	22	9
Sensitivity	86%	100%	100%	100%	78%
True negatives	49	69	62	46	61
Negatives	50	69	63	49	62
Specificity	98%	100%	98%	94%	98%

A total of 20 strains have predicted resistance to at least one beta-lactam antibiotic, and 16 are predicted to possess resistance to multiple beta-lactam antibiotics assessed (Fig. 3). The most highly resistant strains were two identical serotype 19A strains, 1103–1 (NP swab) and 1103–2 (MEF), samples from the same individual who was a part of the AOM with ruptured TM or requiring tympanocentesis cohort. These isolates have predicted resistance to the full panel of beta-lactam antibiotics assessed (when including intermediate levels), as well as tetracycline, erythromycin, and clindamycin. However, strains with predicted resistance to multiple beta-lactam antibiotics predominantly occurred in a cluster of genomes containing serotype 35B strains. All 35B strains showed a similar resistance profile, with many also having predicted resistance to erythromycin, with the exception of strain 1113 which is predicted to have heightened (complete) resistance to amoxicillin and meropenem as well. Finally, a high degree of resistance was detected in a single NP isolate (1001 from the URI cohort) representing a divergent, and non-typable SPN lineage, with a resistance profile similar to that of isolate 1113 (35B serotype from the AOM with ruptured TM or requiring tympanocentesis cohort).

AMR profiling for “penam” resistance was also performed using CARD [38], but it only identified 5 isolates with relevant penicillin-binding protein (PBP) gene mutations, 4 of which did have clinical SPN MIC values >  = 0.25 for penicillin (Additional File 1: Table S6). Of note are hits from contigs of isolates 1056 and 1070–2 that were not identified as being of SPN origin. Isolate 1056 had a very short fragment of *mexB* that appeared to come from *Pseudomonas* and isolate 1070–2 had short fragments of the genes for KPC-73, TEM-91, OmpA, and *E. coli* soxS with a mutation conferring antibiotic resistance with possible *E. coli* and/or *K. pneumoniae* origins. As these hits are so short, they are not reliable predictors of antibiotic resistance, however, the non-SPN specific method for AMR profiling may have picked up on alternate sources of penicillin resistance in the clinical tests where, for isolate 1070–2, no SPN AMR source could be predicted.

#### Phylogenetic placement of untypable sample “1001”

**Fig. 4:**
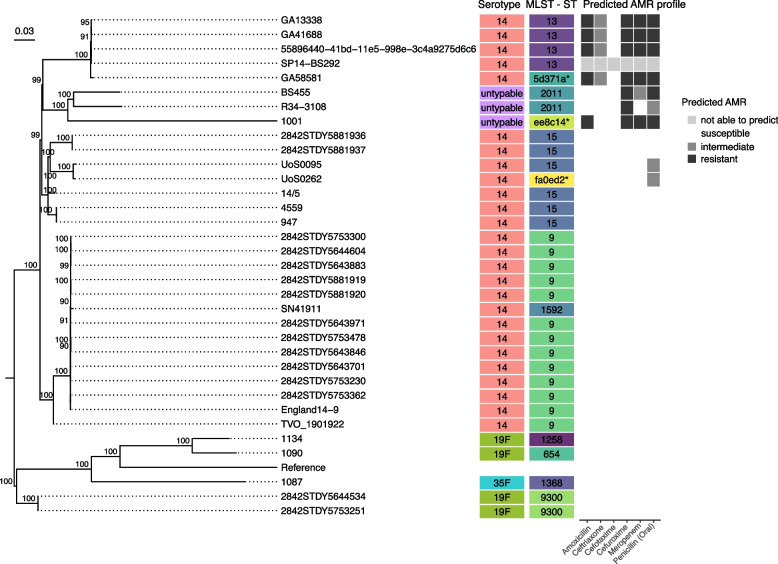
Comparison of a novel SPN strain to its closest relatives in the NCBI-Genbank database. The top 30 SPN genomes in NCBI by average nucleotide identity compared to novel isolate 1001, as well as its closest related isolates from this study: 1087, 1134, and 1090. The Snippy alignment for this genome-based tree was created using SPN D39V (NCBI accession # NZ_CP027540.1) as a reference (serotype, MLST-ST, and predicted AMR profile left blank). The mid-point rooted tree is visualized with the ggtree() package in R and only bootstrap values greater than 80 were visualized on the tree. MLST, serotyping, and the AMR profile data are to the right of the tree (Additional File 1: Table S8). The colours for serotype and MLST sequence type columns are to help differentiate the types

Next, we further analyzed the untypable sample 1001 (in the URI cohort) given its phylogenetic novelty combined with its unique AMR profile. We compared isolate 1001 to its closest SPN genomes by average nucleotide identity from the NCBI-Genbank database (Additional File 1: Table S7). The top 30 closest genomes had a range of 99.00—99.16% average nucleotide identity. In a genome-based phylogeny, 1001 was placed in a well-supported clade with other highly resistant strains (Fig. 4 and Additional File 1: Table S8). SPN strain R34-3108 is from a nasopharynx sample of a patient in Massachusetts in 2004. The individual was 6–24 months old with no listed symptoms. BS455 is from a nasopharynx of a patient from Pennsylvania in 1999. The patient had a middle ear infection (with pink eye and ear pulling). The branch length between these two strains and isolate 1001 is quite large, possibly indicating an accumulation of mutations over the 15–30 year difference in sample collection. Virulence factor analysis with VFanalyzer revealed an extreme depletion in capsule gene matches of the untypable strains (1001, R34-3108, and BS455), compared to Virulence Factor Database SPN representative strains D39 and CGSP14 (Additional File 1: Table S9). 1001 and BS455 also had a lack of matches to the choline-binding protein genes *pspA* and *pspC*/*cbpA*, but had extra matches to *lytA*.

### WGS analysis of HFLU isolates

**Fig. 5:**
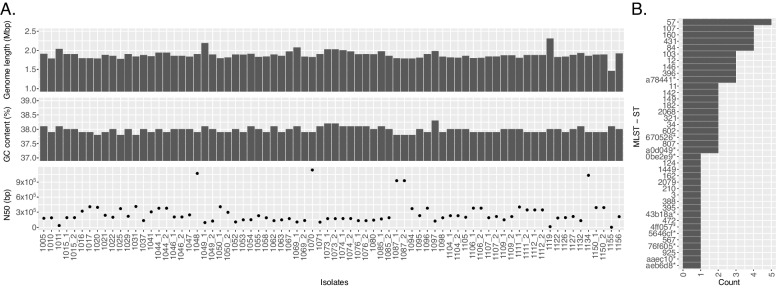
WGS assembly statistics and MLST for 76 HFLU isolates. **A** The assembly statistics shown here are from Pathogenwatch. Data in Additional File 1: Table S11. Isolates with “-1” or no “-” came from NP swabs while isolates with “-2” came from MEF samples, apart from 1071, which is from a MEF sample. **B** MLST sequence type frequency across the 76 isolates. Any MLST classification considered “untypable” are indicated here by the first six characters of their classification followed by an asterix. Full classification strings can be found in Additional File 1: Table S12

A total of 76 HFLU isolates were identified from clinical samples, corresponding to paired NP swab and MEF samples from 58 children aged 6–35 months (Additional File 1: Table S1). As with the SPN isolates, we assembled genomes, filtered the contigs based on taxonomic profiling, and performed analysis using Pathogenwatch (Fig. 5 and Additional File 1: Tables S10 and S11). All assemblies were verified as HFLU by MLST analysis. However, a few assemblies (1085_1, 1155, and 1011) had high amounts of non-HFLU profiled DNA indicative of contamination (51.14% *S. capitis*, 53.91% *M. catarrhalis*, and 44.30% *H. haemolyticus*, respectively). Similar findings have been reported in previous WGS studies of HFLU cultures and attributed to possible mixed populations or laboratory contamination [31]. There was enough HFLU DNA left in the assemblies to have a genome size comparable to the NCBI HFLU complete genome average of 1.88 +—0.06 Mbp), except for 1155 which ended up being smaller at 1.46 Mbp and is very likely incomplete (Fig. 5A). When considering all the isolates, the genome assembly statistics were very close to expected values, with an average size of 1.88 +—0.10 Mb and a GC content of 37.98 +—0.10% (compared to the NCBI HFLU complete genome GC content average of 38.11 +—0.10%) (Fig. 5A).

#### Phylogenetics and strain typing of HFLU isolates

MLST analysis identified 29 different sequence types, with the most common (57) present in 5 isolates (Fig. 5B and Additional File 1: Table S12). A total of 14 isolates were untypeable, but some still had consistent classifications across NP swab and MEF sample pairs (e.g. 1112_1 and 1112_2). Typing based on the cap locus revealed all but isolate 1070 to be nontypable, with isolate 1070 predicted to be serotype F encapsulated.

**Fig. 6:**
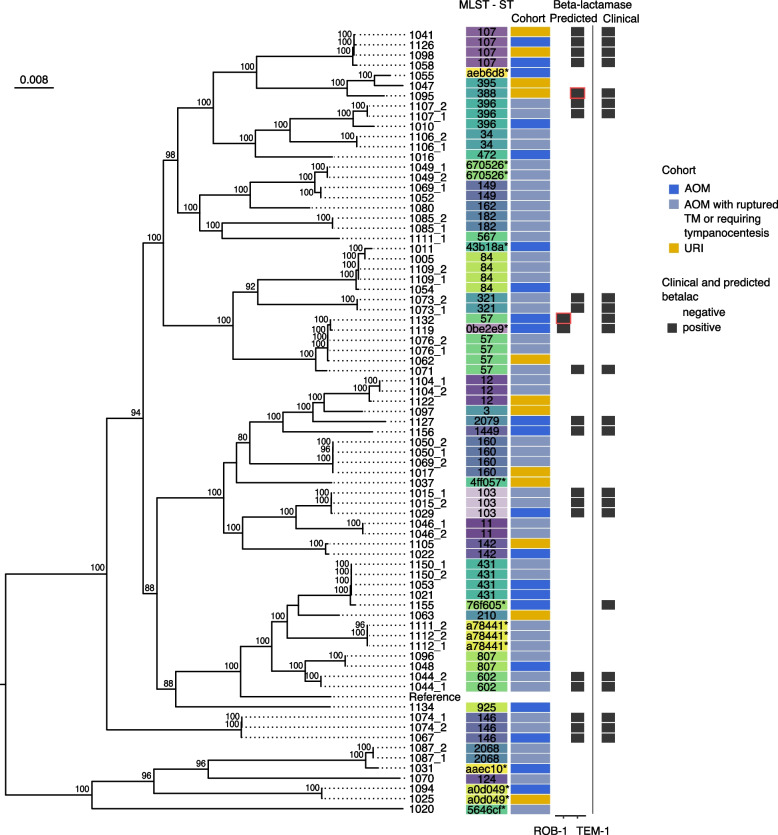
Genome-based phylogeny of HFLU isolates. A Snippy alignment was created using HFLU strain 65290-NP-Hi3 (NCBI accession # NZ_QWLX01000001) as a reference against the 76 HFLU isolates. The mid-point rooted tree is visualized with the ggtree() package in R, with its bootstrap support greater than 80 displayed for each applicable node. MLST, beta-lactamase predictions, and clinical metadata were left blank for the reference. Predictions outlined in red were added back in due to isolate 1132 having a gene split across either end of a linearized plasmid and isolate 1095 having a contig incorrectly assigned to *H. parainfluenzae*. Isolates with “-1” or no “-” came from NP swabs while isolates with “-2” came from MEF samples, apart from 1071, which is from an MEF sample. Colours for the MLST sequence type column are only present to help differentiate between the types

A genome-based phylogenomic tree of the 76 HFLU strains was constructed with HFLU strain 65290_NP_Hi3 (NCBI accession # NZ_QWLX01000001) as a reference genome. The HFLU isolates have a large diversity of well-supported lineages, similar to the SPN isolates (Fig. 6). All NP swab and MEF sample pairs cluster together in the tree, except for those from samples 1069 and 1111, and the MLST sequence types cluster along clade divisions, with few exceptions (type 57 having one untypable isolate, 1119, in the clade). However, as with the SPN isolates, there was no clustering apparent for the patient cohort type.

#### AMR profiling of HFLU isolates and comparison with clinical results

To perform WGS-based AMR profiling, since this feature is not yet implemented in Pathogenwatch for HFLU, we analyzed all 76 genomes for the presence of the beta-lactamase gene using CARD [36]. Twenty isolates were predicted to have a beta-lactamase gene (a ROB, or TEM beta-lactamase) using the “Perfect” search paradigm with the most common being the TEM beta-lactamase (TEM-1: 19 isolates, Fig. 6 and Additional File 1: Table S13). The calculated profile matches the clinically tested beta-lactamase presence test results very well, with only three isolates, 1095, 1155, and 1132, having a positive beta-lactamase test but not having a predicted match. Isolate 1155, which was heavily contaminated with *M. catarrhalis*, had a perfect match to the BRO-1 sequence on a contig that was taxonomically profiled as belonging to *M. catarrhalis*, which was not considered in the original AMR profile as it did not come from HFLU. Isolate 1095 has a perfect match to the TEM-1 sequence on a contig identified by Kraken 2 as belonging to *H. parainfluenzae*. A BLASTN analysis of this contig found top matches at 99.70% identity to nine HFLU strains including P652-8881 and 11P6H (NCBI accession #s CP031684.1 and CP020014.1, respectively) and as such was added back into the 1095 HFLU genome. As for isolate 1132, the other isolate in its clade, 1119, has a *bla*_ROB_ gene match and when the relatively less exacting “Strict” search results were considered, strain 1132 was found to have two 100% identity matches to each half of the ROB-1 beta-lactamase sequence profile. The two partial ROB-1 sequences were found to reside on a putative plasmid-derived contig and assembled as a complete ROB-1 sequence when the plasmid contig was circularized. This contig has near perfect BLASTN matches (99.97% ID) to HFLU strain BB1059 plasmid PB1000, strain F50 plasmid pB1000, and strain BB1052 plasmid pB1000 (NCBI accession #s HM470204.1, HM236408.1, and GU080064.1, respectively).

Most of the predicted beta-lactamase genes were *bla*_TEM-1_ with three isolates having predicted *bla*_ROB-1_ genes, as described above. As of Jul. 22, 2022, CARD shows that ROB-1 sequences are present in some strains of *Pasteurella* and *Haemophilus*. It is present in only 0.23% of 692 HFLU whole-genome shotgun assemblies sequences tested (and in 0% of the 95 completely sequenced genomes scanned), being a relatively rare gene for HFLU. On the other hand, *bla*_TEM-1_ is a much more common gene, found in a large variety of Gram-negative species, present in 12.43% of the HFLU genome sequences tested (and 10.53% of the completely sequenced genomes). The *M. catarrhalis* contaminated isolate 1155 was the only isolate with a match to BRO-1*.* CARD shows that BRO-1 sequences only seem to be present in *M. catarrhalis*, and as a follow-up we used BLASTN to compare the contig containing the *bla*_BRO-1_ beta-lactamase gene to the NCBI nt database. The contig from isolate 1155 is a perfect match to *Moraxella catarrhalis* strain MC8 and strain 142P87B1 (NCBI accession #s CP010902.1 and CP034665.1, respectively). As the isolate with this *bla*_BRO-1_ gene prediction, 1155, tested positive for beta-lactamase presence, it is likely the positive result was due to *Moraxella* contamination in this assembly. When not considering this isolate 1155, the sensitivity for CARD detecting HFLU penam antibiotic resistance was 100% with the specificity also reaching 100%. Thus, the agreement between WGS-based AMR profiling and clinical testing was perfect, and even higher than that achieved for SPN AMR profiling.

## Conclusions

In this work, we provide a new dataset of 148 SPN and HFLU genomes obtained from a pediatric AOM and URI cohort, and we also outline a WGS-based workflow using a combination of bioinformatics tools that rapidly and accurately assigned taxonomy and AMR profiles in excellent agreement with clinical laboratory phenotyping results. Our work contributes to ongoing genomics-based studies of clinical SPN and HFLU infectious disease samples, which is important to track the emergence and diversification of new pathogen variants, and inform ongoing vaccine design strategies and AMR surveillance efforts [53–56].

Although many infectious disease laboratories have transitioned to WGS-based methods for pathogen characterization (e.g., MLST, serotyping, AMR profiling), some challenges we faced when exploring such methods in our clinical use case were as follows: 1) which bioinformatic methods to choose for accurate typing and AMR-profiling of HFLU and SPN isolates from pediatric AOM/URI samples? 2) How accurate are these methods in comparison to standard non-WGS methods? Our study aimed to address these questions using an original dataset of samples obtained from pediatric AOM/URI cases. To our knowledge, this is the first study of its kind that applies and assesses the accuracy of pathogen genomics and AMR profiling tools (Pathogenwatch and CARD) tools on SPN and HFLU isolates from pediatric AOM cases. Our study therefore outlines a bioinformatic workflow based on a combination of easy-to-use tools and web-servers that could be adopted in clinical microbiology workflows, and forms the groundwork for future larger-scale studies of pediatric HFLU and SPN isolates that are currently underway.

For SPN, we show that the Pathogenwatch automated pipeline was able to verify taxonomy for 71/72 suspected SPN isolates, agreeing with the assembly taxonomic profiling done through Kraken 2, together re-identifying one isolate as the related species, *S. mitis*. The AMR profiling method for SPN penicillin susceptibility was also shown to be highly accurate as it had a 95% agreement with clinical tests, a 2% false positive rate, and a 14% false negative rate. For other antibiotics including ceftriaxone, tetracycline, erythromycin, and clindamycin, it also produced results that were in strong agreement with clinical testing (mean sensitivity of 95% and a mean specificity of 98%; Table 2). In addition to the use of WGS in AMR profiling, genomic analysis also identified potentially rare lineages of SPN, such as a divergent SPN isolate (1001) from a patient with an URI, most closely related to other nasopharyngeal samples including isolates from 1999 and 2004.

For HFLU, we found that a combined workflow involving both Pathogenwatch, hicap, and CARD was effective for typing and AMR profiling. CARD’s beta-lactamase predictions showed remarkable agreement with clinical tests for beta-lactamase presence with beta-lactamase genes found in all isolates with a positive beta-lactamase clinical test.

Although there was overall strong agreement between WGS-based predictions and traditional clinical microbiological testing, in several cases WGS analysis identified differences in the taxonomic identity of cultured isolates and impurities in cultured isolates that likely affected clinical test results. One example of this was the *bla*_ROB-1_ gene detected in a suspected HFLU isolate with high amounts of *M. catarrhalis* contig contamination and a positive beta-lactamase clinical test. Genomic analysis revealed that this *bla*_ROB-1_ gene was likely of *Moraxella catarrhalis* origin. Another example is SPN isolate 1070–2, which had a positive clinical AMR test but did not have predicted resistance by WGS-based AMR profiling. Further investigation of this sample revealed putative AMR gene fragments from potential contaminant organisms including *E. coli,* which could have resulted in a false positive clinical test. These examples highlight the ability of WGS to refine the interpretation of clinical diagnostic results, and its importance in ongoing surveillance efforts for characterizing AOM/URI pathogens.

### Supplementary Information

**Additional file 1: Table S1.** Clinical metadata. **Table S2.** Kraken 2 contig classifications weighted by contig length (% total assembly size) for *S. pneumoniae* isolates. **Table S3.** Assembly statistics from Pathogenwatch for *S. pneumoniae* isolates. **Table S4.** Serotype, MLST sequence typing, AMR profile, and select clinical metadata for *S. pneumoniae* isolates. **Table S5.** Additional AMR predictions compared to clinical metadata for *S. pneumoniae* isolates. **Table S6.** Penam resistance hits from CARD results. **Table S7.** FastANI results for *S. pneumoniae* isolate 1001 compared to NCBI-Genbank *S. pneumoniae* strains. **Table S8.** Sequence type, serotype and AMR profile for *S. pneumoniae* isolate 1001's closest related *S. pneumoniae* strains. **Table S9.** VFDB comparison of virulence factor genes across select *S. pneumoniae* strains. **Table S10.** Kraken 2 contig classifications weighted by contig length (% total assembly size) for *H. influenzae* isolates. **Table S11.** Assembly statistics from Pathogenwatch for *H. influenzae* isolates. **Table S12.** MLST sequence typing and select clinical metadata for *H. influenzae* isolates. **Table S13.** Beta-lactamase hits from CARD results.

**Additional file 2: Figure S1.** Comparison of a novel *S. mitis* strain “1015” to its closest *S. mitis* relatives in the NCBI-Genbank database. **Table S1.** FastANI results for *S. mitis* isolate 1015 compared to NCBI-Genbank *S. mitis* strains. **Table S2.** VFDB comparison of virulence factor genes across select *S. mitis* strains.

## Data Availability

The datasets supporting the conclusions of this article are available under the BioProject ID PRJNA946631 in the SRA repository [SRR23912767-911 and SRR23913123-5] and within the article’s additional files.

## Abbreviations

AOM: Acute otitis media
AMR: Antimicrobial resistance
CARD: Comprehensive antibiotic resistance database
DNA: Deoxyribonucleic acid
HFLU: *H. influenzae*
MEF: Middle ear fluid
MLST: Multilocus sequence typing
NP: Nasopharyngeal
PBP: Penicillin-binding protein
PCR: Polymerase chain reaction
SPN: *S. pneumoniae*
TM: Tympanic membrane
URI: Upper respiratory infection
WGS: Whole genome sequencing
