# Supplementary material for: Genomic classification and antimicrobial resistance profiling of Streptococcus pneumoniae and Haemophilus influenzae isolates associated with paediatric otitis media and upper respiratory infection
Source: BMC Infect Dis. 2023 Sep 13;23:596. doi: 10.1186/s12879-023-08560-x (PMC10498559; doi:10.1186/s12879-023-08560-x)
Supplement: Supplementary file 2 — Additional file 2: Figure S1. Comparison of a novel S. mitis strain “1015” to its closest S. mitis relatives in the NCBI-Genbank database. Table S1. FastANI results for S. mitis isolate 1015 compared to NCBI-Genbank S. mitis strains. Table S2. VFDB comparison of virulence factor genes across select S. mitis strains. [file 12879_2023_8560_MOESM2_ESM.pdf]

Additional File 2

Genomic investigation of a novel S. mitis strain

We also further investigated the "outlier" strain "1015" since WGS analysis identified this is an *S. mitis* strain instead of an SPN strain as initially classified by culture-based identification. Of 173 *S. mitis* genomes in NCBI, the top 10 closest strains by average nucleotide identity ranged from 94.96 – 95.57% with the highest average nucleotide identity being from strain SK137 (GCA_000960025.1, **Additional File 2: Table S1**). A SNP-based tree confirmed the *S. mitis* placement in a clade with strain SK137 (amongst a few other *S. mitis* strains). While VFanalyzer did not have an *S. mitis* representative genome, comparison with other *S. mitis* strains (NCTC_12261, CR124, SK145, and SK137) showed a lack of matches to the pneumococcal iron uptake gene *piuA* (**Additional File 2: Table S2**). Otherwise, the virulence factor genes outlined by VFanalyzer had match congruence across the *S. mitis* strains compared.

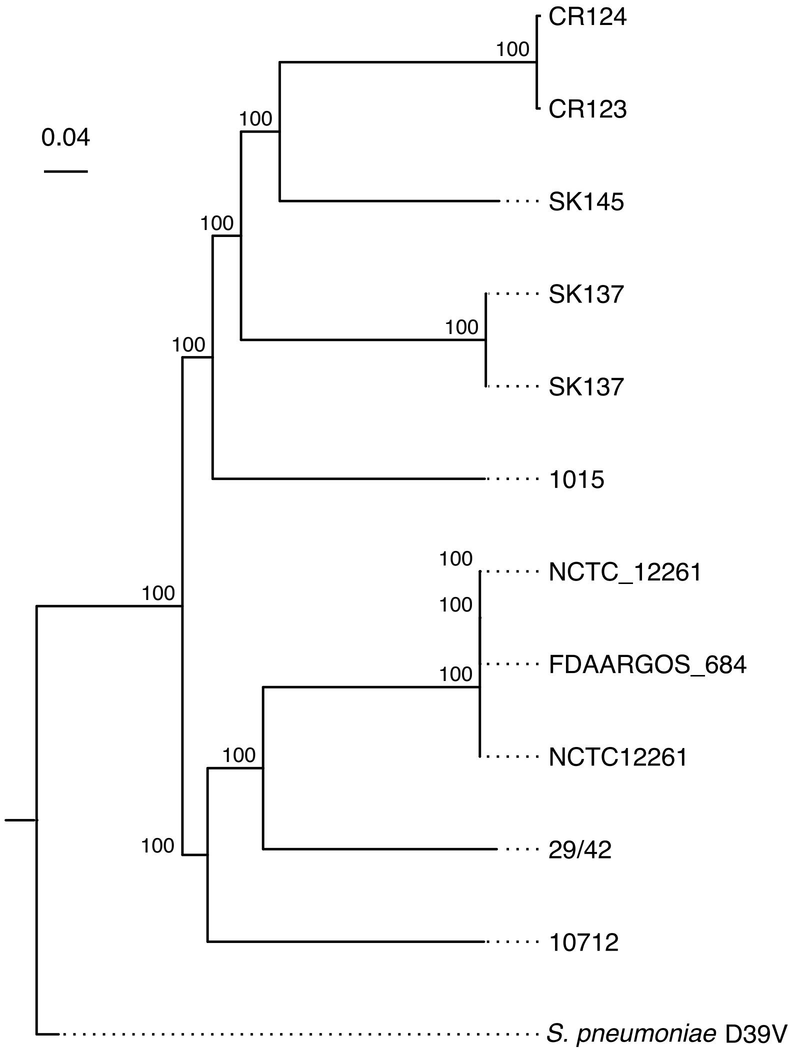

Additional File 2: Figure S1. Comparison of a novel *S. mitis* strain "1015" to its closest *S. mitis* relatives in the NCBI-Genbank database. These 10 strains had the highest average nucleotide identity to isolate 1015 out of 173 genomes in NCBI. The average nucleotide identity values and assembly IDs are available in **Additional File 2: Table S1**. The phylogenetic tree was genome-based using a Snippy alignment (reference was *S. mitis* strain NCTC 12261, NCBI accession # NZ_CP028414.1) and created with RaxML using automated bootstrap convergence criteria and the GTR+GAMMA substitution model. The tree was mid-point rooted and visualized with the ggtree() package in R.

Additional File 2: Table S1. FastANI results for *S. mitis* isolate 1015 compared to NCBI-Genbank *S. mitis* strains

Genome assembly ID	Strain	ANI (%)	Count of bidirectional fragment mappings	Total query fragments
GCA_000960025.1	SK137	95.5658	566	648
GCA_000722835.1	SK137	95.5392	554	648
GCA_000960065.1	SK145	95.5338	581	648
GCA_019929545.1	CR123	95.0381	522	648
GCA_019929615.1	CR124	95.004	536	648
GCA_000430365.1	29/42	94.9972	549	648
GCA_001588625.1	10712	94.9789	544	648
GCA_000148585.3	NCTC 12261	94.9766	539	648
GCA_900459425.1	NCTC12261	94.9656	548	648
GCA_009730515.1	FDAARGOS_684	94.9625	549	648

Additional File 2: Table S2. VFDB comparison of virulence factor genes across select *S. mitis* strains

VFclass	Virulence factors	Related genes	*S. pneumoniae* D39 (serotype 2)	NCTC_12261	CR124	SK145	SK137	1015
			chromosome (NC_008533)	chromosome (NZ_CP028414.1)	draft (GCA_019929615)	draft (GCA_000960065.1)	draft (GCA_000960025)	draft
	Choline-binding proteins	cbpD	SPD_2028	orf01706	orf00498	orf00471	orf01818	orf01291
		cbpG	SPD_0356*	-	-	-	-	-
		lytA	SPD_1737	-	orf00896	-	orf00908	-
		lytB	SPD_0853	orf00780	orf01022	orf01145	orf00559	orf00545
		lytC	SPD_1403	orf01292	orf00301	orf00026	orf01327	orf01528
		pce/cbpE	SPD_0821	orf00754	orf00997; orf00999	-	-	orf00570
		pspA	SPD_0126	-	-	-	orf01133	orf01487
		pspC/cbpA	SPD_2017	-	-	-	-	-
		pavA	SPD_0854	orf00781	orf01023	orf01146	orf00560	orf00544
	Laminin-binding protein	lmb	SPD_0888	orf00932	orf00543	orf01507	orf00742	orf00362
	Sortase A	srtA	SPD_1076	orf00818	orf00555	orf01358	orf00599	orf00504
	Streptococcal lipoprotein rotamase A	slrA	SPD_0672	orf00656	orf00143	orf01011	orf00432	orf00666
	Streptococcal plasmin receptor/GAPDH	plr/gapA	SPD_1823	orf00180	orf00441	orf01343	orf01296	orf01820
		hysA	SPD_0287	-	-	-	-	-
	Neuraminidase A	nanA	SPD_1504	-	-	-	-	-
	Streptococcal enolase	eno	SPD_1012	orf00946	orf00531	orf01542	orf00754	orf00326

**Immune evasion**	Capsule	Undetermined	SPD_0315; SPD_0316; SPD_0317; SPD_0318; SPD_0319; SPD_0320; SPD_0321; SPD_0322; SPD_0323; SPD_0324; SPD_0325; SPD_0326; SPD_0327; SPD_0328	orf00133; orf01432; orf01435; orf01441; orf01442; orf01443; orf01444; orf01445; orf01446; orf01447; orf01679	orf00390; orf00391; orf00392; orf00393; orf00394; orf00418	orf00189; orf00190; orf00191; orf00192; orf00193; orf00443; orf01295	orf01250; orf01471; orf01480; orf01481; orf01482; orf01483; orf01484; orf01790	orf01021; orf01022; orf01023; orf01024; orf01025; orf01264; orf01354
**Iron uptake**	Pneumococcal iron acquisition	piaA	SPD_0915	-	-	-	-	-
	Pneumococcal iron uptake	piuA	SPD_1652	orf00279	orf01650	orf00616	orf00088	-
**Manganese uptake**	Pneumococcal surface antigen A / Metal binding protein SloC	psaA	SPD_1463	orf00487	orf01747	orf00849	orf00272	orf00830
**Protease**	C3-degrading protease	cppA	SPD_1278	orf01093	orf01182	orf01704	orf00895	orf00175
	IgA1 protease	iga	SPD_1018	-	-	-	-	-
	Serine protease	htrA/degP	SPD_2068	orf01741	orf00779	orf00506	orf01852	orf01326
	Trigger factor	tig/ropA	SPD_0365	orf01410	orf01548	orf00160	orf01448	orf00993
	Zinc metalloproteinase	zmpB	SPD_0577	-	-	orf01834	orf01071	-
		zmpC	-	orf00074	-	orf01234	orf01184	orf01439
	Pneumolysin	ply	SPD_1726	-	-	-	-	-
**Antiphagocytosis**	Capsule(Enterococcus)	cpsI	-	-	orf00404	orf00179	-	orf01012

Table saved from VFDB (http://www.mgc.ac.cn/VFs/) [Tue Mar 29 19:55:56 2022 - Sat Apr 2 00:47:39 2022].
